# Immunotherapy in gynecological cancers

**DOI:** 10.37349/etat.2021.00033

**Published:** 2021-02-28

**Authors:** Domenica Lorusso, Valentina Ceni, Gennaro Daniele, Antonella Pietragalla, Vanda Salutari, Margherita Muratore, Camilla Nero, Francesca Ciccarone, Giovanni Scambia

**Affiliations:** 1 Division of Gynecologic Oncology, Fondazione Policlinico Universitario Agostino Gemelli IRCCS, Largo Agostino Gemelli 8, 00168 Rome, Italy; 2 Department of Life Science and Public Health, Università Cattolica del Sacro Cuore, 00168 Rome, Italy; 3 Department of Gynecology and Obstetrics, University of Parma, 43126 Parma, Italy; Università Politecnica Marche, Italy

**Keywords:** Immunotherapy, ovarian cancer, endometrial cancer, cervical cancer

## Abstract

Immunotherapy has changed the natural history of several malignancies that, a decade ago, had a very poor prognosis, such as lung cancer and melanoma. Consequently, many attempts have been done to expand the indications of immunotherapy agents, predominantly immune checkpoint inhibitors (ICIs), in other cancers, including gynecological malignancies. Alongside promising results in cervical and endometrial neoplasms, there are not clear data on the benefit of ICIs as single agent or in combination with antiangiogenic agents in ovarian cancer (OC) and ongoing trials are focusing on combining ICIs with standard chemotherapy or PARP inhibitors. This chapter summarized the evidences of ICIs in gynecological malignancies and report the ongoing trials in cervical, endometrial and OC.

## Introduction

In the last decade immunotherapy has revolutionized the course of several cancers, such as lung, melanoma, and urogenital cancers.

Immunotherapy essentially acts by boosting the endogenous immune system against tumor cells. Active immunotherapy works by stimulating the host’s own immune system *versus* malignant cells through cancer vaccines, such as dendritic peptide-, cell-, RNA-, DNA-vaccines or oncolytic viruses. Passive immunotherapy consists in administering immune compounds produced exogenously, stimulating an anti-tumor immune response. The latter, includes immune checkpoint inhibitors (ICIs) and adoptive T cell therapy (ACT) [1].

To elude the immune system, tumor cells grow a locally immunosuppressed microenvironment, that down-regulate T cells activation by creating inhibitory pathways: these latter exploit cytotoxic T-lymphocyte-associated protein 4 (CTLA-4), and programmed death-ligand 1 (PD-L1) and its receptor [programmed death-1 (PD-1)/PD-L1]. CTLA-4 acts on regulatory T cells binding competitively B7.1 and B7.2 ligands expressed on antigen-presenting cells (APCs), and consequently preventing their binding with stimulatory receptor CD28: this eventually blocks the activation of cytotoxic T lymphocytes. The T cell activation regulation by CTLA-4 mainly occurs within secondary lymphoid organs [2].

PD-1 is a molecule expressed on the surface of activated T and B cells, which binds to PD-L1 and PD-L2: the first ligand, PD-L1, is expressed on non-hematopoietic cells, leukocytes and in non-lymphoid tissues; the second ligand, PD-L2, is expressed, instead, on monocytes and dendritic cells [3]. This different expression pattern of PD-L1 and PD-L2, and B7 molecules just described is behind the hypothesis that CTLA-4 acts first to induce tolerance, whereas PD-1 joins later for maintaining long-term tolerance [4].

Tumor cells, overexpressing PD-L1 and PDL-2, develop the capacity to promote PD-1 signaling in tumor-infiltrating CD4 and CD8 T cells, therefore creating a locally immunosuppressed microenvironment [2]. Thus, inhibiting the PD-1/PD-L1 pathway reinstates the immune response against tumors and constitutes the rationale for using immune checkpoint blockade agents (ICIs) in tumor management; some examples of PD-1 or PD-L1 agents are: pembrolizumab, nivolumab, dostarlimab and cemiplimab or atezolizumab, avelumab and durvalumab, respectively.

Differently, ACT consists in the reinfusion, into cancer patients, of lymphocytes (T cells), either allogenic or autologous, after *in vitro* activation and expansion. While such treatment won’t induce immunological memory, it grants immediate protection, albeit in the short-term only. Several types of ACT therapies are available: tumor-infiltrating lymphocytes (TILs), natural killer (NK) cells, lymphokine-activated killer (LAK) cells, and, more recently, T cell receptors (TCRs) and chimeric antigen receptors (CARs), able to target a wide range of potential cellular types. TCRs are obtained from the patient’s lymphocytes transfected with a viral vector carrying *TCR* genes, and may identify antigens derived specifically from a tumor-reactive T cell clone. CARs are modified membrane receptors, composed of an extracellular antibody single-chain variable fragment (scFv), a TCR-derived internal domain, and one or more intracellular co-stimulatory domains [5].

## Immunotherapy in cervical cancer (CC)

Persistent HPV infection is the recognized cause of almost all CCs. HPV escapes the immune system in different ways: down regulation of MHC class I (by HPV-E7 protein), impairment of antigen presentation (by HPV-E5 protein), expression of immune suppressive factors and, most importantly, attracting immune cells that can inhibit the immune response through the indoleamine-pyrrole 2,3-dioxygenase (IDO) enzyme [6]. Several studies reported elevated rates of PD-1/PD-L1 expression in up to 80% of cervical tumors: hence, supporting immunotherapy could be decisive to restore the immune response against tumor in CC.

PD-1 inhibitor pembrolizumab (MK-3475) was firstly studied in KEYNOTE-028, a phase Ib trial enrolling 475 patients with PD-L1–positive advanced solid tumors [including cervical, endometrial and ovarian cancer (OC)]. Patients were administered pembrolizumab at a dose of 10 mg/kg every two weeks until disease progression or unacceptable toxicity or for a maximum of 2 years [7]. Twenty-four patients joined the CC cohort: overall response rate (ORR) was found to be 17% with a median duration of response (DoR) of 5.4 months and 13% of patients experienced stable disease (SD). Five patients reported grade 3 treatment-related adverse events (AEs) mainly consisting in cutaneous rush and proteinuria. No grade 4 AEs or deaths were observed [8].

In the same trial, 13% ORR was reported in the 24 patients with advanced endometrial cancer (EC) [9] and 11.5% ORR in the 19 patients with advanced OC [10].

Pembrolizumab was further investigated in KEYNOTE-158, a phase II, non-randomized, multi-cohort trial. Ninety-eight patients with recurrent or metastatic CC were treated with pembrolizumab 200 mg q3w, until unacceptable toxicity or disease progression. At a median follow-up of 10.2 months, ORR was 12.2%, with three complete responses (CRs) and nine partial responses (PRs). The latter responses occurred in patients with PD-L1–positive tumors where 14.6% ORR was registered and the median DoR was not reached; 65.3% of patients experienced treatment-related AEs (12.2% grade ≥ 3), mainly hypothyroidism (10.2%), decreased appetite (9.2%), and fatigue (9.2%) [11]. Based on this data, in June 2018 the FDA approved pembrolizumab for the treatment of advanced/recurrent, previously treated, CPS ≥ 1 CC, with CPS being the ratio of PD-L1 staining cells (both tumor cells, and immune cells) to the total number of viable tumor cells × 100.

Nivolumab is another PD-1 inhibitor that has been tested in HPV-related malignancies. In the CheckMate 358 trial nivolumab (240 mg every 2 weeks) was tested in a population of heavily pretreated cervical, vulvar and vaginal cancer patients: the disease control rate (DCR) was 70.8% and the ORR was 20.8% (responses to therapy were only noted in the CC cohort with a ORR 26.3%) [12].

The results of CheckMate 358 study, testing two combination regimens of nivolumab plus ipilimumab, a monoclonal antibody targeting CTLA-4, in advanced CC, were presented at ESMO Congress in 2019. Ninety-one patients with recurrent squamous cervical tumors, with or without prior systemic therapies, were randomly chosen to receive nivolumab at 3 mg/kg q2weeks plus ipilimumab at 1 mg/kg q6weeks (Nivo3 + Ipi1) or nivolumab at 1 mg/kg plus ipilimumab at 3 mg/kg, given q3w for 4 doses followed by nivolumab at 240 mg q2w (Nivo1 + Ipi3). In the Nivo1 + Ipi3 arm ORRs were higher both in the chemo-naive patients (46% *vs.* 32%) and in patients with prior systemic therapy (36% *vs.* 23%). Similarly, median overall survival (OS) was 25.4 and 10.3 months with Nivo1 + Ipi3 and Nivo3 + Ipi1, respectively, in pretreated patients while was not reached in the chemo-naive population [13].

Besides the trials above mentioned (Table 1), ongoing trials are exploring the role of ICIs combined with chemo-radiation CX-11 [14], or neo-adjuvant chemotherapy MITO CERV 3 [15], in locally advanced disease, and in combination with systemic platinum-based chemotherapy in the advanced setting, either in chemo-naïve patients (KEYNOTE-826 [16] and BEAT-CC trial [17]) or in previously treated patients (CX-8 GEMAB [18] and CX-9 REGENERON trial [19]).

**Table 1.** Clinical trials in CC

**Author**	**Trial/Phase**	**Setting**	**Pts *N***	**Treatment**	**Results**	**Grade 3–4 AEs Pts *N* (%)**
Lheureux et al., 2015 [25]	NCT01693783 Phase I-II	Metastatic, recurrent	42	Ipilimumab 10 mg/kg q3w for 4 cycles If CR/PR/SD 4 → cycles ipilimumab 10 mg/kg q12w every 12 weeks	ORR 8.8%	-diarrhea *n* = 4 (9.5) -colitis *n* = 3 (7.1)
Chung et al., 2019 [11]	KEYNOTE-158 Phase II	PD-L1 positive advanced	98 (82, PDL1 CPS ≥ 1)	Pembrolizumab 200 mg q3w	ORR 12.2% 14.6% in PD-L1 positive Total population: Median PFS 2.1 Mo. Estimated PFS rate at 6 Mo. 25.0% Median OS 9.4 Mo. 6-month estimates OS 75.2% 12-month estimates OS 41.4% PD-L1 positive: Median PFS 2.1 Mo. Median OS 11 Mo. 6-month estimates OS 80.2% 12-month estimates OS 47.3%	Treatment related: -increased ALT *n* = 3 (3.1) -increased AST *n* = 2 (2.0) Immune-mediated: -hepatitis *n* = 2 (2.0) -severe skin reactions *n* = 2 (2) -adrenal insufficiency *n* = 1 (1)
Wendel Naumann et al., 2019 [12]	CheckMate 358 Phase I-II	HPV-associated tumors, recurrent or metastatic cervical, vaginal, vulvar cancers	24 (19 cervical, 5 vaginal-vulvar cancer)	Nivolumab 240 mg q2w	ORR: 26.3% (cervical) 20.0% (vaginal-vulvar) DCR: 68.4% (cervical) 80.0% (vaginal-vulvar) Median PFS 5.1 Mo. 26.3% progression free patients at 12 Mo. In cervical cohort: Median OS 21.9 Mo., 12-month OS rate 77.5% 24-month OS rate 49.8%	Cervical cohort: -diarrhea *n* = 1 (5.3) -hepatocellular injury *n* = 1 (5.3) -pneumonitis *n* = 1 (5.3) Vaginal/vulvar cohort: none

Pts *N*: patient number; PFS: progression free survival; Mo.: month

Given the immunomodulatory effects of bevacizumab, ICIs have also been combined with antiangiogetic drugs in order to capture a possible synergist effect of the association in advanced CC, where antiangiogenetic drugs are already licensed. The BEAT trial is investigating the combination of bevacizumab-cisplatin-paclitaxel and atezolizumab in the first line CC metastatic disease.

In the context of ACT, Stevanović et al. [20], reported an ORR of 33% after a single infusion of E6 and E7 reactive TIL and IL-2 (after lympho-depletion chemotherapy) in a population of heavily pretreated, advanced CC; these promising results call for further investigation.

Finally, thanks to the success of prophylactic HPV vaccines in the prevention of cervical dysplasia, there is great drive to develop therapeutic HPV vaccines targeting E6 and E7 oncoproteins. The most promising of these appears to be the ADXS11-001, amalimo-gene-filolisbac: a live attenuated *Listeria monocytogenes* (*Lm*) vaccine containing E7 oncoprotein from HPV-16, ADXS11-001 is under evaluation in different trials, both as single agent (GOG/NRG-0265 [21]) or in combination with Durvalumab [22] or cisplatin [23].

Unfortunately, the results of the above mentioned trials are not strong enough to be cost-effective, especially in undeveloped countries where CC ranks second in incidence and its mortality is only behind breast cancer [24].

## Immunotherapy in EC

EC patients are diagnosed at early-stage disease with a 5-year OS of 80%, advanced stage patients present a dismal prognosis with 5-year survival of about 15% [26].

According to the Tumor Cancer Genome Atlas (TCGA) classification, EC subtypes with high tumor mutational burden, POLE mutant and microsatellite instability hypermutated (MSI-H) with a deficiency in the mismatch repair system (dMMR), are highly immunogenic: that is, they exhibit elevated tumor specific neoantigens, resulting in an enlarged number of CD3^+^ and CD8^+^ TILs and in a compensatory up regulation of immune checkpoints [27]. MSI-H is found in approximately 30% of EC [28], 40–80% of endometrioid, 10–68% of serous, and 23–69% of clear cell EC, which overexpressed PD-1/PD-L1, suggesting that the targeting of this pathway may constitute a hopeful strategy to increase antitumor immune response [29].

The FDA approval of pembrolizumab in solid tumors with MSI-H or dMMR, was “tumor agnostic” and based on data across five uncontrolled, multi-cohort, single-arm trials: a total of 149 patients with MSI-H were identified and in the 14 patients cohort of EC the ORR was 36% [30].

In September 2019 FDA approved the combination of pembrolizumab plus lenvatinib for the treatment of advanced EC non harboring MSI-H, based on the results of KEYNOTE-146, a single-arm study in advanced EC patients progressing after at least one prior systemic chemotherapy line. Patients were treated with lenvatinib 20 mg orally once per day in combination with pembrolizumab 200 mg every 3 weeks, until progression of disease. Among 108 enrolled patients, 94 presented microsatellite stable or MSS (not MSI-H or dMMR) stable tumors, 11 had MSI-H tumors, and in 3 patients MSI status was not known. The ORR was 38.3% with 10 CRs (10.6%) and 26 PRs (27.7%) in MSS cohort [31, 32]. EMA indication is pending, waiting for the results of a randomized trial comparing Lenvatininb-pembrolizumab *versus* physician’s choice chemotherapy in advanced setting.

PD-1 inhibitor nivolumab was investigated in a phase II study in a mixed cohort of patients at the dose of 240 mg every 2 weeks. Preliminary results indicated an ORR of 22.7% in the EC cohort: notably, ORR was similar regardless of PD-L1 expression, with all patients with MSI-H tumors experiencing at least a PR [33].

In the GARNET trial, the PD-1 inhibitor dostarlimab (TSR-042) was assessed on a population of 94 advanced ECs at the dose of 500 mg IV every 3 weeks for the first 4 cycles, followed by 1,000 mg IV every 6 weeks thereafter. The ORR was 27.7% (50% in MSI-H tumors and 19.1% in MSS tumors) and the DCR was 48.9% [34]. At an update analysis, among 70 patients with MSI-H tumors, the ORR was 43% (9 patients had a confirmed CR and 21 had a PR) and the DCR was 59% [35].

In a preliminary phase Ia study atezolizumab was administered at the dose of 1,200 mg q3weeks to 15 advanced EC patients reporting an ORR of 13% and a DCR of 26% without significant treatment related AEs; it is worth noting that in tumors presenting both elevated PD-L1 expression and TILs, the ORR appeared higher [36].

Recently, the combination of durvalumab with or without tremelimumab, a CTLA-4 inhibitor, was examined in 56 recurrent EC patients (NCT03015129). In the durvalumab monotherapy arm the ORR was 14.8% with 13.3% of patients not progressing at 24-week, while in the combination arm the ORR was 11.1% with a 24-week PFS of 18.5%. Grade 3–4 treatment related AEs were 11% and 43% in the monotherapy and combination arm, respectively [37].

Besides the trials above mentioned (Table 2) there is a variety of ongoing trials evaluating the role of ICIs in EC either as single agents (NCT02899793 [38] and NCT02912572 [39]) or in combination with other ICIs [40] or chemotherapy in advanced disease, both in chemonaive (LEAP trial [41], AtTEND trial [42], Ruby trial [43], MITO END 3 [44]) or pretreated patients (NRG-GY018 [45], KEYNOTE-775 [46]).

**Table 2.** Clinical trials in EC

**Author**	**Trial/Phase**	**Setting**	**Pts *N***	**Treatment**	**Results**	**Grade 3–4 AEs Pts *N* (%)**
Marabelle et al., 2020 [47]	KEYNOTE-158 Phase II	MSI-H/dMMR relapsed pretreated	49	Pembrolizumab 200 mg q3w for 2 years	ORR 57.1% Median DoR NR (2.9 to 27.0+)	Treatment related: -fatigue *n* = 2 (0.9) -asthenia *n* = 1 (0.4) Immune-mediated: -hyperthyroidism *n* = 1 (0.4) -pneumonitis *n* = 3 (1.3) -colitis *n* = 2 (0.9) -hepatitis *n* = 2 (0.9) -severe skin reactions *n* = 3 (1.3) -diabetes mellitus 1 *n* = 1 (0.4) -Guillain-Barre syndrome *n* = 1 (0.4) -pancreatitis *n* = 1 (0.4)
Makker et al., 2019 [31]	KEYNOTE-146 Phase II	Metastatic, no more than two prior systemic therapies	53	20 mg oral lenvatinib daily plus 200 mg pembrolizumab q3w	ORR 39.6% investigator review ORR 45.3% independent review	Treatment-related: Grade 3: *n* = 36 (68) Grade 4: none Most common: -hypertension *n* = 18 (34) -diarrhoea *n* = 4 (8) -palmar-plantar erythrodysesthesia syndrome *n* = 3 (6)
SGO 2019 Annual meeting [48]	GARNET Phase I	Previously treated recurrent or advanced	110	Dostarlimab 500 mg q3w for 4 cycles → 1,000 mg q6w	ORR 27.7% (50.0% in MSI-H; 19.1% in MSS) DCR 48.9%	Treatment-related: Grade 3–4 *n* = 13 11.8% Most common: -AST increased (2.7)

Pts *N*: patient number; Mo.: month; NR not reached

## Immunotherapy in OC

PD-L1 seems to be particularly expressed in OC cells compared to other neoplasms and it is known to result in worse survival. Moreover, having a high frequency of TILs and neoantigen load in select groups of patients, such as those presenting homologous recombination deficiency (HRD) or MSI-H OC represents a promising target for immunotherapy, therapeutic vaccines and ACT [49]. Nevertheless, no immunotherapy agent is yet approved in OC, so far, due to the unsatisfactory results from ICIs in any setting of disease.

KEYNOTE-100, a phase II trial, enrolling two cohorts of relapsed OC patients (*n* = 376): cohort A recruiting women previously pretreated with 1–3 lines of chemotherapy with a treatment-free interval (TFI) of 3–12 months; and cohort B including patients with up to 6 prior lines and a TFI longer than 3 months. In the overall population the ORR was 8% and the DCR was 37%; considering the two cohorts separately, ORR was 7.4% in cohort A and 9.9% in cohort B. Median PFS was 2.1 months in the overall population, and median OS was 18.7 months in cohort A and 17.6 months in cohort B (presented at ASCO annual meeting). The expression of PD-L1, evaluated using CPS score, showed an ORR of 5.0%, in patients with CPS < 1, whereas it was 10.2% if CPS ≥ 1 and 17.1% if CPS ≥ 10 [50].

In JAVELIN 200 trial 566 platinum resistant OC patients were randomized to receive pegylated liposomal doxorubicin (PLD), avelumab or the combination of both. Unfortunately, the combination of avelumab and PLD did not extend the median PFS (3.5 months *vs.* 3.7 months) and the median OS (13.1 months *vs.* 15.7 months) with respect to PLD single agent [51].

In a single arm phase II study 37 platinum resistant patients treated with the combination of weekly paclitaxel and pembrolizumab, reported an ORR of 51.4%, a DCR of 86.5%, with 6-month progression free in 64.5% of patients, a median PFS of 7.6 months and a median OS of 13.4 months [52].

The possibility of combining different ICIs was investigated in the NRG-GY003 trial: 100 patients with recurrent OC were randomized to receive nivolumab alone (arm 1), or the combination of nivolumab and ipilimumab, followed by nivolumab maintenance (arm 2). The platinum-free interval (PFI) stratified hazard ratio (HR) for PFS was 0.528 and the HR for death was 0.789 [53].

Despite the negative results in the recurrent setting, ICIs were investigated in first line treatment, based on the hypothesis that a less exhausted immune system in chemo naïve patients could possibly translate in a better efficacy of strategy.

Avelumab (Ave) was investigated in a phase III randomized trial with Ave and chemotherapy (CT), JAVELIN 100 trial, both in combination and maintenance (CT + Ave → Ave) or only as maintenance after carboplatin-paclitaxel (CT → Ave) in newly diagnosed, advanced OC; the control arm consisted in patients receiving placebo as maintenance (CT → O).

The study was prematurely terminated for futility at a median follow-up of 11 months; at the pre-planned interim analysis the PFS HR in CT → Ave arm *vs.* control was 1.43 and in CT + Ave → Ave arm *vs.* control was 1.14. The ORR were 30.4%, 36.0% and 30.4% for CT → Ave, CT + Ave → Ave and CT → O arms respectively, with a significant increase in toxicity in both experimental arms [54].

Due to the deluding results of immunotherapy administered as monotherapy, researchers have explored the possibility of combining ICIs with parp inhibitors and antiangiogenic agents.

While HRD tumors are characterized by an elevated PD-L1 expression, probably because of the persistence of not lethal DNA defects that continuously stimulate innate immune cell to release pro-inflammatory substances, they are likely to escape immune control, switching from a Th1-immunity to a chronic inflammation and immunosuppression. Poly (ADP-ribose) polymerase inhibitors (PARPi), triggering a catastrophic DNA damage, especially in HRD cells, could, therefore, reestablish a productive Th1 immune response, thus readjusting the tumor microenvironment [55]. In BRCA mutated mouse models, PARPi increased the mutational tumor load and TILs and activated interferon-mediated pathway by synergizing with ICIs, consequently providing a strong case in favor of drug combinations [56].

The combination of a PARPi, olaparib, with a PD-L1 inhibitor, durvalumab, was the objective of investigation of a multi-cohort trial, called MEDIOLA. Platinum-sensitive recurrent OC patients’ cohort, with a known or suspected deleterious germline BRCA1/2 mutation, received olaparib 300 mg bid and durvalumab 1,500 mg every four weeks: the ORR was 71.9% (7 CRs) with a median DoR of 10.2 months and a 28-week DCR of 65.6%; median PFS was 11.1 months and median OS was not reached, with 87.0% of patients alive at 24 months [57].

The TOPACIO/KEYNOTE-162 trial explored, in women with advanced or metastatic triple-negative breast cancer (TNBC) or recurrent OC, irrespective of *BRCA* mutation status, the combination of pembrolizumab and niraparib. Among the 60 patients of the OC cohort, the ORR was 18% (3 CRs and 8 partial responses), with a DCR of 65% and a median DoR not reached (range, 4.2 to ≥ 14.5 months); remarkably the ORR was found to be uniform among subgroups, divided in patients with platinum sensitive OC, pre-treated with Bevacizumab and BRCAmut or HRD [58].

Many ongoing clinical trials are exploring the potential advantages of combining PARPis with ICIs and antiangiogenic drugs in newly diagnosed OC (AGO DUO [59], ENGOT OV 43 [60], FIRST [61] and ATHENA trial [62], MITO 28 [63]), in combination and maintenance after first line carboplatin-paclitaxel chemotherapy.

The rationale for combining immunotherapy with antiangiogenic agents lies on the ability of the latter to enhance T cell trafficking and infiltration into the tumor microenvironment [64]: in preclinical models, the inhibition of vascular endothelial growth factor (VEGF) signaling promoted antitumor immunity and enhanced the efficacy of immune checkpoint blockade [65], moreover the combination of anti-VEGF and anti-PD-L1 showed a synergistic anti-tumor effect *in vivo* [66]. This combination is under investigation both in first line and in platinum-sensitive and platinum-resistant relapses.

IMagyn050/GOG 3015/ENGOT-OV39 is a phase III randomized study evaluating the administration of atezolizumab/placebo in combination with carboplatin-paclitaxel-bevacizumab in 1,300 newly-diagnosed, stage III-IV OC patients [67].

The failing results of this trial were presented at the last ESMO Congress in 2020: PFS in the intent-to-treat population (1,301 patients) was 19.5 months with the combination atezolizumab/bevacizumab/ chemotherapy *versus* 18.4 months with only bevacizumab and chemotherapy (HR, 0.92). In the PD-L1–positive population (*n* = 784), the median PFS was 20.8 months *vs.* 18.5 months with the atezolizumab regimen and standard treatment respectively (HR, 0.80). However, results from exploratory PFS analyses demonstrated a trend supporting atezolizumab in patients with PD-L1 immune cells of 5% or greater. Future studies could investigate whether better patients’ selection is associated with increased outcomes.

Besides the trials above mentioned (Table 3), the combination of atezolizumab and bevacizumab has been testing in two other ongoing trials in the recurrent setting: NCT03353831 [68] and NCT02839707 [69].

**Table 3.** Clinical trials in epithelial OC, EOC

**Author**	**Trial/Phase**	**Setting**	**Pts *N***	**Treatment**	**Results**	**Grade 3–4 AEs Pts *N* (%)**
Matulonis et al., 2019 [50]	KEYNOTE-100 Phase II	Advanced recurrent Cohort A: 1-3 prior lines PFI/ TFI 3-12 Mo. Cohort B: 4-6 prior lines PFI/ TFI ≥ 3 Mo.	Cohort A: 285 Cohort B: 91	Pembrolizumab 200 mg q3w until 2 years	Total population: ORR 8%, DCR of 37% Cohort A: ORR 7.4%, DoR 8.2 Mo. DCR 37.2% OS NR Cohort B: ORR 9.9% DoR NR DCR 37.4% OS 17.6 Mo.	Treatment-related: -fatigue *n* = 10 (2.7) -anemia *n* = 5 (1.3) -colitis *n* = 5 (1.3) Immune-mediated: -severe skin reactions *n* = 7 (1.9) -colitis *n* = 6 (1.6)
SGO 2020 Annual meeting [72]	JAVELIN 100 Phase III	First line	998	6 cycles carboplatin AUC 5/6, q3w, + paclitaxel 175 mg/mq q3w or 80 mg/mq weekly a) + avelumab 10 mg/kg q3w (with CT)/q2w (maintenance) b) + avelumab 10 mg/kg q2w maintenance c) no avelumab	ORR： a) 36.0% b) 30.4% c) 30.4%	Grade 3–4 AEs a) 70.8% b) 66.5% c) 62.6%
NCT02580058 [51]	JAVELIN 200 Phase III	Platinum resistant	566	a) PLD 40 mg/mq q4w b) Ave 10 mg/kg q2w c) combination of both	PFS: a) 3.5 Mo. b) 1.9 Mo. c) 3.7 Mo. OS: a) 13.1 Mo. b) 11.8 Mo. c) 15.7 Mo. (HR, 0.89)	Abdominal pain: a) 3.39% b) 4.81% c) 3.30% Intestinal obstruction: a) 3.39% b) 5.88% c) 4.95% Vomiting: a) 1.69% b) 3.74% c) 2.20%
Drew et al., 2019 [57]	MEDIOLA Phase II	BRCA-mutated platinum-sensitive relapsed	32	Olaparib monotherapy 300 mg bid 4 weeks → olaparib 300 mg bid + durvalumab 1,500 mg IV q4w	ORR 71.9% 28-wk DCR 65.6% Median PFS 11.1 Mo. Median DoR 10.2 Mo.	-anaemia 17.6% -elevated lipase 11.8% -neutropenia 8.8% -lymphopenia 8.8% Discontinuation due to AEs: Olaparib 5 pts Durvalumab 3 pts
Konstantinopoulos et al., 2019 [58]	TOPACIO Phase I-II	Recurrent platinum-resistant	60	Niraparib 200 mg daily + pembrolizumab 200 mg q3w	ORR 18% DCR 65%	Treatment-related: -anemia *n* = 11 (21) -thrombocytopenia *n* = 5 (9) -leukopenia *n* = 3 (6) -neutropenia *n* = 2 (4) Immune-related AEs: *n* = 3 (6)

Pts *N*: patient number; Mo.: month; NR not reached

The first one is a phase III, multicenter, randomized trial evaluating the safety and efficacy of combining bevacizumab plus atezolizumab and chemotherapy, compared to bevacizumab plus placebo and chemotherapy in the relapsed disease (first and second recurrence within 6 months after platinum containing chemotherapy); the second trial is a phase II/III study assessing the combination of atezolizumab/ bevacizumab with PLD.

The combination of nivolumab (anti-PD1) and bevacizumab (anti-VEGF) has been studied in a phase II trial in 38 recurrent OC heavily pre-treated (≥ 3 chemotherapy lines) patients. In the overall population the objective response rate was 28.9% (40% *vs.* 16.7% in the platinum-sensitive and resistant setting, respectively); the median PFS was 9.4 months (12.1 and 7.7 months in sensitive and resistant patients, respectively). Thirty-four patients (89.5%) revealed at least one treatment-related adverse event and 23.7% a grade 3 or higher treatment-related adverse event [70].

Cediranib, an oral VEGF receptor inhibitor, has been evaluated in combination with durvalumab, an anti-PDL1 inhibitor, in the recurrent OC setting, in heavily pre-treated patients. A phase I trial reported a DCR of 75% with six partial response and three stabilizations of disease. Grade 3 and 4 AEs occurred on the daily schedule, those were: pulmonary embolism (2/8), hypertension (2/8), diarrhea (2/8), lymphopenia (1/8) and pulmonary hypertension (1/8) while only hypertension (1/6) and fatigue (1/6) were present at grade 3 and 4 in the intermittent cediranib schedule. The investigators concluded that the association of durvalumab plus intermittent cediranib is tolerable and clinically active [71].

## Conclusion

Immunotherapy represents a potentially promising strategy to expand the therapeutic armamentarium of gynecological malignancies and many trials are going in cervical, endometrial and OC (Table 4).

**Table 4.** Clinical trials on ICIs in gynecological malignancies

**Disease**	**Setting**	**ICIs**		
		**CTLA-4**	**PD-1**	**PD-L1**
		**Ipilimumab** **Tremelimumab**	**Pembrolizumab** **Nivolumab** **Dostarlimab** **Cemiplimab**	**Ave** **Atezolizumab** **Durvalumab**
OC	NACT first-line		ENGOT OV 43 [60] FIRST [61] ATHENA [62] MITO 28 [63]	AGO DUO [59]
	ROC Plat Se	NRG-GY003 [53]	NCT02873962 [77]	ANITA [79] ATALANTE [80]
	ROC Plat R		NCT02440425 [52] NCT02873962 [74]	EORTC 1508 [81] NCT02484404 [82]
EC	Advanced (III-IV) recurrent CT naive		LEAP-001 [41] NRG-GY018 [45]	MITO END-3 [44] ATTEND [42] RUBY [43]
	Recurrent prior CT	NCT03015129 [37] NCT02982486 [40] NCT02834013 [73]	NCT02899793 [38] KEYNOTE-775 [46] NCT02549209 [45]	MITO END-3 [44] ATTEND [42] NCT02912572 [39] NCT03526432 [83]
CC	LACC	NCT01711515 [74]	MITO CERV 3 [15] NCT04221945 [14]	
	Recurrent metastatic		NRG-GY002 [75] KEYNOTE-826 [16] REGENERON [19] CX 8 [76]	NCT02921269 [78] BEAT [17]

NACT: neoadiuvant chemotherapy; ROC: recurrent epithelial ovarian cancer; Plat Se/Plat R: platinum sensitive/resistant; LACC: locally advanced cervical cancer

In CC, clinical data on the use of immunotherapy either as monotherapy, or in combination with chemotherapy, radiotherapy and other ICIs, appear promising; ongoing studies will better address the best setting in which this strategy will produce the higher benefit.

In EC the available data are conflicting, with some studies suggesting higher benefit in MSI-H and POLE mutated tumors, some others reporting efficacy regardless the molecular profile of the disease, especially when ICIs are combined with oral tyrosine-kinases inhibitors with antiangiogenic properties. Ongoing trials in advanced, chemo-naïve patients, combining immunotherapy with platinum-base chemotherapy, will possibly change the standard of care in this setting.

Immunotherapy as single agent and in combination with antiangiogenic agents reported disappointing results in any setting of OC disease, mostly because of the unavailability of a reliable predictive biomarker of response and, as a consequence, a bias in patient selection. Our expectations and hopes are that the ongoing trials studying the combination of ICIs with PARPis will clarify the role, if any, of immunotherapy in OC treatment.

## Abbreviations

ACT: adoptive T cell therapy
AEs: adverse events
Ave: Avelumab
CARs: chimeric antigen receptors
CC: cervical cancer
CPS: combined positive score
CR: complete response
CTLA-4: cytotoxic T-lymphocyte-associated protein 4
DCR: disease control rate
dMMR: deficient mismatch repair system
DoR: duration of response
EC: endometrial cancer
HRD: homologous recombination deficiency
ICIs: immune checkpoint inhibitors
MSI-H: microsatellite instability hypermutated
OC: ovarian cancer
ORR: overall response rate
OS: overall survival
PARPi: poly (ADP-ribose) polymerase inhibitor
PD-1: programmed death-1
PD-L1: programmed death-ligand 1
PFI: platinum-free interval
PFS: progression free survival
PLD: pegylated liposomal doxorubicin
PR: partial response
SD: stable disease
TCRs: T cell receptors
TFI: treatment-free interval
TILs: tumor-infiltrating lymphocytes

## Declarations

### Copyright

© The Author(s) 2021.
